# P-543. Role of respiratory viral infections in Airflow Obstruction after Pediatric Hematopoietic Cell Transplantation

**DOI:** 10.1093/ofid/ofaf695.758

**Published:** 2026-01-11

**Authors:** Sapna Pardasani, Ali Y Suliman, Jose Amadeo A Ferrolino, Ronald H Dallas, Megan Peterson, Pamela Merritt, Amanda Cole, Amber Davis, Ashleigh Gowen, Kim J Allison, Randall Hayden, Ying Li, Dinesh Keerthi, Saumini Srinivasan, Gabriela Maron, Brandon Triplett, Diego R Hijano

**Affiliations:** St. Jude Children's Research Hospital, Dallas, TX; St. Jude Children's Research Hospital, Dallas, TX; St. Jude Children's Research Hospital, Dallas, TX; St. Jude Children's Research Hospital, Dallas, TX; St. Jude Children's Research Hospital, Dallas, TX; St. Jude Children's Research Hospital, Dallas, TX; St. Jude Children's Research Hospital, Dallas, TX; St. Jude Children's Research Hospital, Dallas, TX; St. Jude Children's Research Hospital, Dallas, TX; St. Jude Children's Research Hospital, Dallas, TX; St. Jude Children's Research Hospital, Dallas, TX; St. Jude Children's research Hospital, Memphis, Tennessee; St Jude Children’s Research Hospital, Memphis, Tennessee; University of Michigan, Ann Arbor, Michigan; St. Jude Children's Research Hospital, Dallas, TX; St. Jude Children's Research Hospital, Dallas, TX; St. Jude Children's Research Hospital, Dallas, TX

## Abstract

**Background:**

Respiratory Viral Infections (RVIs) in allogeneic hematopoietic cell transplant (allo-HCT) recipients can cause airflow obstruction (AFO), leading to pneumonia or death, particularly in those with preexisting conditions. Despite this risk, the impact of community-acquired RVI on PFTs in pediatric patients after HCT remains unclear.

**Figure:**
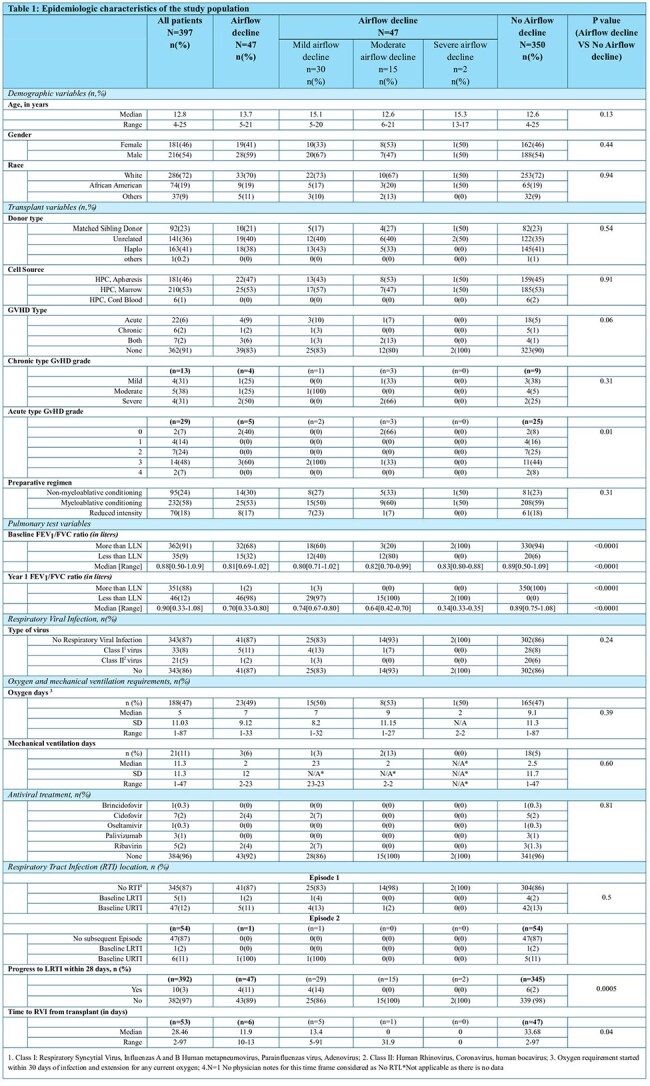

**Figure:**
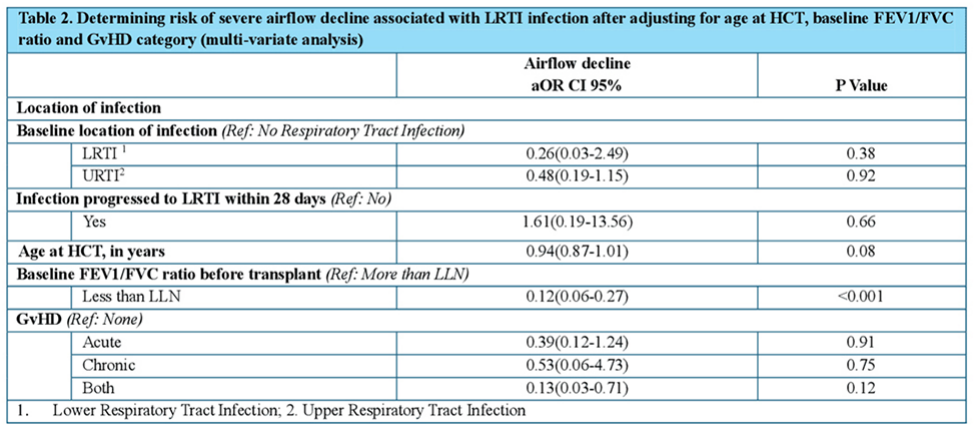

**Methods:**

This retrospective study analyzed St. Jude patients undergoing HCT between 2003-2020. Data from electronic health records included patient demographics, transplant information, microbiological results, and RVI episode information during 100 days post-HCT. AFO was defined by z-scores and FEV1/FVC ratio expressed as LLN. Baseline parameters were obtained before HCT, and Year 1 parameters within 425 days post-HCT. Spirometry measures were calculated using GLI calculator. Bivariate and multivariate logistic regression identified risk factors for airflow decline following HCT.

**Figure:**
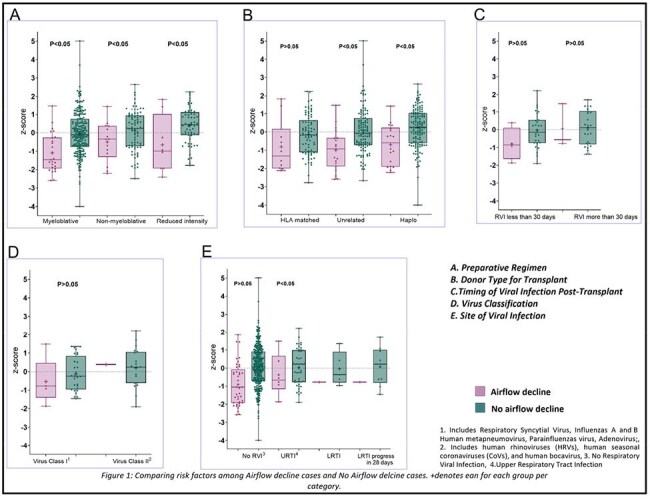

**Figure:**
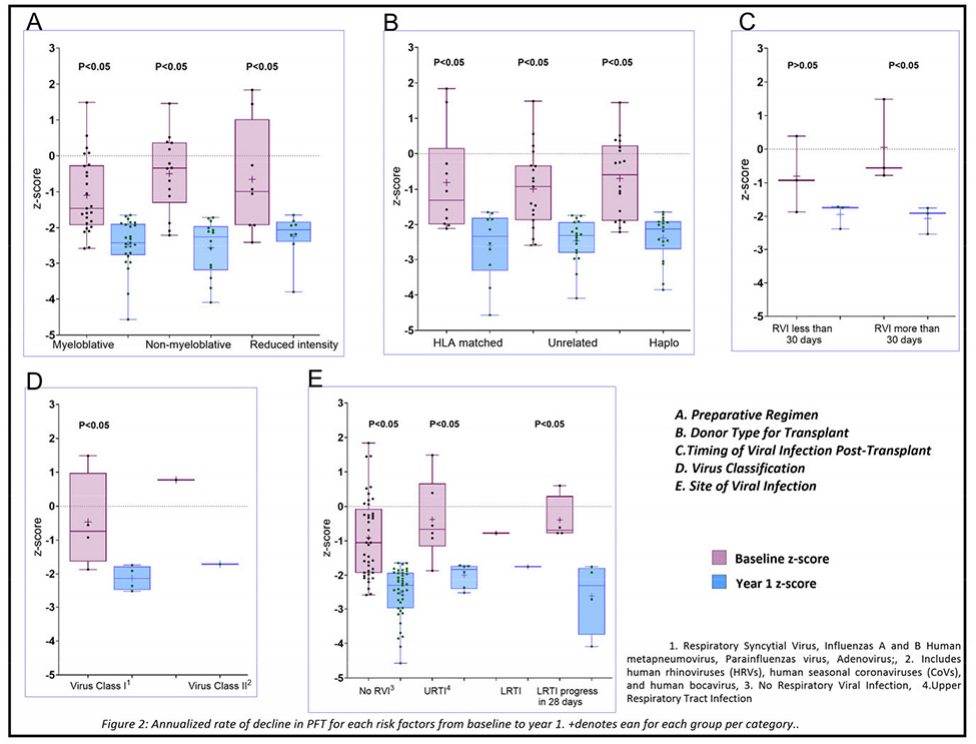

**Results:**

A total of 397 patients were included in the study, with a median age of 12.7 years (Table 1). Approximately 13% developed RVI within the first 100 days post-transplantation. Of these, the majority (78%) required oxygen therapy (median days=7), which was significantly (P< 0.05) longer than that of individuals without viral infections (median days=5). Furthermore, patients with RVI who required oxygen were significantly (P< 0.05) more likely (14% vs 10%) to require ICU admission than those without an infection who did not require oxygen. Among patients with AFO (12%), 68% had a baseline FEV1/FVC ratio above LLN, 11% had a history of Lower Respiratory Tract Infection (LRTI), which was significantly (P< 0.05) higher as compared to those without AFO (2%). The median time from transplant to RVI was significantly shorter in patients with AFO (12 days vs 34 days). We found baseline LLN as a significant predictor for airflow decline (Table 2). However, our model (Table 2) did not find a significant association between the risk of severe airflow decline and RVI after adjusting for covariates which have been identified as risk factors for airflow decline in the literature (Figure 2).

**Conclusion:**

Respiratory viral infection within 100 days of allo-HCT did not significantly affect airflow obstruction following HCT in pediatric patients.

**Disclosures:**

Randall Hayden, MD, Abbott: Board Member|Abbott: Serving on the advisory board|Cepheid: Board Member|Cepheid: Serving on the advisory board|Roche Diagnostics: Advisor/Consultant|Roche Diagnostics: Board Member|Roche Diagnostics: Serving on the advisory board Gabriela Maron, MD, MS, SymBio Pharamaceuticals: Advisor/Consultant|SymBio Pharamaceuticals: Grant/Research Support

